# Supplementary material for: Splice-Junction-Based Mapping of Alternative Isoforms in the Human Proteome
Source: Cell Rep. Author manuscript; Available in PMC 2020 Jan 15. (PMC6961840; doi:10.1016/j.celrep.2019.11.026)
Supplement: 2 [file NIHMS1546469-supplement-2.zip › DF1/PXD009737/TestisTrypsin/TestisTrypsin_34_KIAA1210_NLQVIVEPK.pdf]

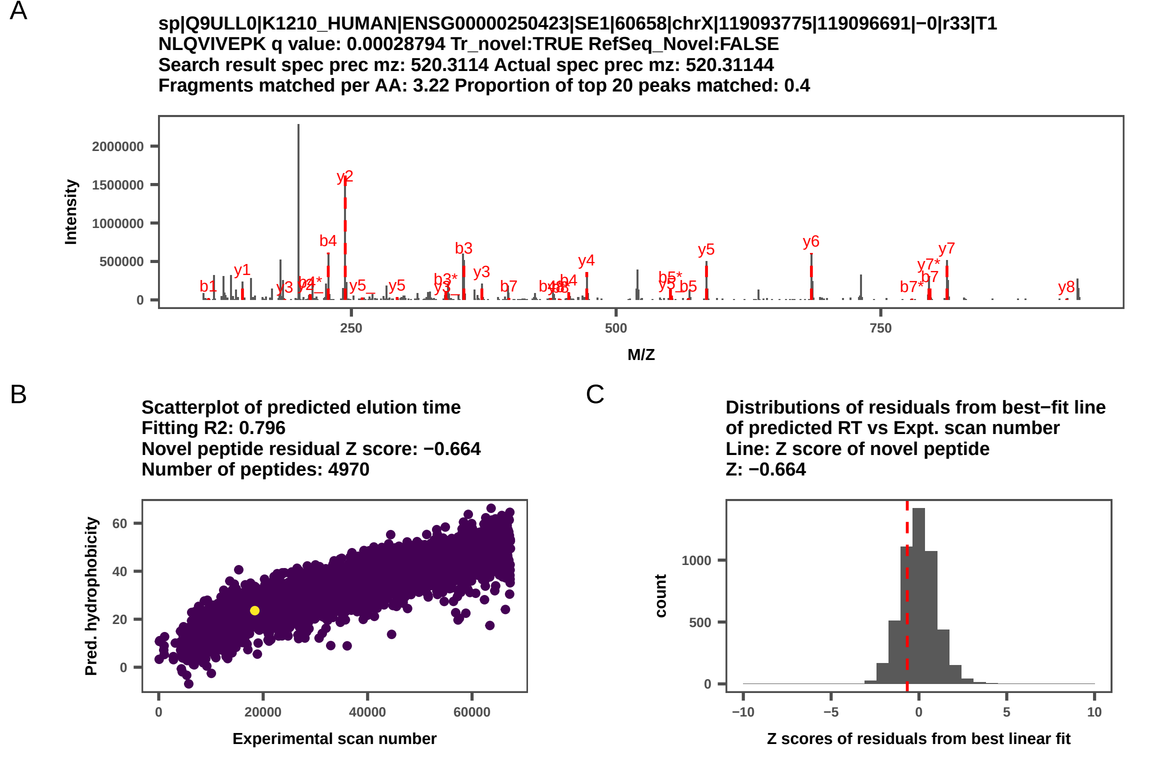

A
sp|Q9ULL0|K1210_HUMAN|ENSG00000250423|SE1|60658|chrX|119093775|119096691|−0|r33|T1
NLQVIVEPK q value: 0.00028794 Tr_novel:TRUE RefSeq_Novel:FALSE
Search result spec prec mz: 520.3114 Actual spec prec mz: 520.31144
Fragments matched per AA: 3.22 Proportion of top 20 peaks matched: 0.4
Intensity
2000000
1500000
1000000
500000
0
y2
b4
b3
y3
y4
y5
y6
y7
y7*
b7
b7*
y8
y1
b1
b7
M/Z
250
500
750
B
Scatterplot of predicted elution time
Fitting R2: 0.796
Novel peptide residual Z score: −0.664
Number of peptides: 4970
Pred. hydrophobicity
60
40
20
0
0
20000
40000
60000
Experimental scan number
C
Distributions of residuals from best−fit line
of predicted RT vs Expt. scan number
Line: Z score of novel peptide
Z: −0.664
count
1000
500
0
−10
−5
0
5
10
Z scores of residuals from best linear fit
